# UK practice in management of patients with poor-grade subarachnoid haemorrhage

**DOI:** 10.1186/cc9744

**Published:** 2011-03-11

**Authors:** H Langrick, C Hammell, E O'Callaghan, G Dempsey

**Affiliations:** 1 University Hospital Aintree, Liverpool, UK

## Introduction

Poor-grade subarachnoid haemorrhage patients have historically fared poorly and often been excluded from aggressive treatment. In a recent audit of practice at our ICU only 33% of these patients were transferred to a neurosurgical centre. Recent studies have demonstrated improved rates of survival with good neurological outcomes in patients receiving rapid resuscitation, control of ICP, early surgery and treatment of cerebral ischaemia [1,2]. We wished to determine national neurosurgical practice with regards to these patients.

## Methods

We conducted a telephone survey of all UK adult neurosurgical centres. We presented the neurosurgical registrar with two mock-up patients - one grade 5 and one grade 4. We asked questions regarding their transfer policy, surgical and medical management, estimated probability of good outcome (Glasgow Outcome Score 4 or 5), and recommendations regarding management if not for transfer.

## Results

None of the 30 units had a policy on whom to transfer. Twenty-one out of 30 (70%) advised transfer of the grade 5 patient and all 30 would transfer the grade 4 patient. Good outcome was estimated at 10% for the grade 5 patient (range <5% to 60%) and 50% for the grade 4 patient (range 20 to 90%). Of those recommending transfer of the grade 5 patient, 12 would proceed to CT angiography and endovascular coiling of the aneurysm within 24 hours. Eight centres would wake and reassess the patient and coil if the GCS improved, seven would place a prophylactic extraventricular drain and nine would routinely insert an intracranial pressure monitor. Of the nine centres that would not transfer, all would subsequently reconsider transfer if GCS improved or hydrocephalus developed. No centres recommended insertion of an intracranial pressure monitor in the referring hospital.

## Conclusions

Treatment of poor-grade subarachnoid haemorrhage remains controversial. In the UK there are no national management guidelines and both recommendations and practice appear to vary considerably between hospitals. Further analysis of national data regarding morbidity and mortality in this patient group is needed. Debate is required to address the question of whether aggressive ICP control is warranted and if so whether this can be provided in a nonspecialist ICU.
